# Special Issue on Agricultural Genebanks

**DOI:** 10.1089/bio.2018.29044.ejp

**Published:** 2018-10-12

**Authors:** Elena Popova

**Affiliations:** Coordinating Editor, Guelph, Canada.

We are fortunate to live in an era when the exceptional importance of conserving the diversity of cultivated plants and their wild relatives is increasingly acknowledged outside the community of its practitioners. “The need to conserve and sustainably use the world's plant genetic diversity is more critical than ever,” stated the Second Global Plan of Action for Plant Genetic Resources for Food and Agriculture, adopted by the FAO Council in 2011.^1^

More recently, the Sustainable Development Goals explicitly link conservation action to development outcome by making clear, with Target 2.5, that the end of hunger and malnutrition depends on maintaining the “genetic diversity of seeds, cultivated plants, farmed and domesticated animals, and their related wild species, including through soundly managed and diversified seed and plant banks at national, regional, and international levels, and ensure access to and fair and equitable sharing of benefits arising from the utilization of genetic resources and associated traditional knowledge as internationally agreed.”^2^

The exceptional worldwide efforts to discover, collect, and research the diversity of cultivated plants in the past century have been crucial to the development of improved varieties of crops that have helped to feed, and raise out of poverty, millions of people. But the widespread adoption of these new varieties has caused the erosion of crop genetic diversity from farmers' fields in many parts of the world. The struggle between losing and discovering valuable gene sources continues, and the ultimate winner of this competition may define the future of humankind.

Crop genebanks, biobanks specializing in the conservation of the genetic diversity of cultivated plant species, find themselves on the frontline of this historic battle. They perform a broad range of functions, from exploring the world for new genetic variants of crops, conserving them for the long term in different forms, carrying out research on them, and documenting their holdings and making them available to users: plant breeders, researchers, farmers, and others who need them. The FAO's Second Report on the State of the World's Plant Genetic Resources for Food and Agriculture suggested that there were 7.4 million accessions in 1750 genebanks in 2010. Even considering the large number of potential duplicates, it is possible that roughly 1.8 million unique accessions are maintained worldwide in national, regional, and international *ex situ* collections of crop diversity.

One of the biggest and most important holdings globally is that managed by the 11 genebanks of CGIAR centers. These centers conserve and make available under the access and benefit sharing arrangements of the Multilateral System of the International Treaty on Plant Genetic Resources for Food and Agriculture (ITPGRFA) >700,000 accessions of crop, tree, and forage germplasm, and are coordinated by the CGIAR Genebank Platform, a partnership between CGIAR and the Global Crop Diversity Trust.

Alongside their basic task of ensuring conservation and availability, these genebanks are working toward achieving the long-term strategic goals of assessing the overall diversity, and the gaps, in their collections, and finding the most effective and efficient methods to deliver to their users the diversity they need. Modern technologies such as rapid phenotyping and genotyping platforms enhance the capacities of genebanks to perform their work but also raise questions as to how this deluge of information should be managed and shared.

This special issue, is based on the experience of the Global Crop Diversity Trust and its partners in building a global system for *ex situ* conservation of crop diversity. It provides a glimpse of how modern agricultural biobanks, including the international genebanks of CGIAR, organize their work to embrace new challenges, and work together to share ideas and technologies.

The Global Crop Diversity Trust is an international organization devoted solely to ensuring the *ex situ* conservation and availability of crop diversity worldwide. It is an essential funding element of the ITPGRFA, an agreement that currently includes 144 countries. To fulfill its mission, the Crop Trust supports genebanks around the world as well as the Svalbard Global Seed Vault, in which seeds originating from almost every country on earth are safely stored beneath the Arctic permafrost. Half a billion dollars has now been invested by governments and the private sector in the work of the Crop Trust since 2004, including in an endowment fund that provides long-term funding to key globally significant genebanks.

This special issue opens with a research article from the International Rice Research Institute and the International Institute of Tropical Agriculture. The authors present evidence that standard conditions for seed processing may not be optimal for subsequent seed longevity and raise a discussion on using alternative approaches to increase storability in some crops.

The review presented by the International Potato Center (CIP) and the International Center for Agricultural Research in the Dry Areas explores various ways of identifying valuable traits in accessions by combining molecular, computer modeling, and classical approaches. The topic is further unfolded by experts from CIP, the International Center for Tropical Agriculture and Bioversity International, who discuss the current and potential use of digital sequence information in conserving and using crop diversity, and its policy implications.

Experts from two countries share their experiences in building national networks of plant genetic resources conservation following two different models: decentralized in Brazil (Embrapa) and centralized in Russia (VIR). Both systems have proved their effectiveness in their respective countries.

The Centre for Pacific Crops and Trees of the Pacific Community in Fiji provides insight into conserving and sharing taro genetic resources in the Pacific. The review illustrates the critical role of a regional genebank in securing, improving, and promoting new genetic diversity of a vegetatively propagated staple food crop for better adaptation to emerging biotic and abiotic stresses.

The authors from Botanic Gardens Conservation International give a bird's-eye view of the historical development of botanic gardens and suggest potential collaboration with agricultural genebanks. The review highlights botanic gardens' profound experience in public outreach and educational programs, which make them a perfect platform to increase public awareness of the importance of plant genetic resources.

Finally, a note by authors from the Nordic Genetic Resource Center and the Crop Trust outlines the global efforts to conserve the duplicates of seed accessions in the Svalbard Global Seed Vault. The number of accessions deposited in the Vault passed 1 million during the 10th anniversary in February 2018.

We hope that the special issue, while not covering all the possible activities of agricultural biobanks, provides a useful overview for biobanking professionals who may not be familiar with the agricultural context, and poses some thought-provoking questions for further discussion. But most of all, we hope it offers an interesting read for all who are interested in where our food ultimately comes from, and how.
